# Electrical Evoked Potentials After Perioperative Pain Neuroscience Education or Back School Education: A Subgroup Analysis of a Randomized Controlled Trial

**DOI:** 10.3390/jcm15010398

**Published:** 2026-01-05

**Authors:** Lisa Goudman, Eva Huysmans, Wouter Van Bogaert, Iris Coppieters, Kelly Ickmans, Jo Nijs, Ronald Buyl, Maarten Moens

**Affiliations:** 1 STIMULUS Research Group, Vrije Universiteit Brussel, Laarbeeklaan 103, 1090 Brussels, Belgium; 2 Department of Neurosurgery, Universitair Ziekenhuis Brussel, Laarbeeklaan 101, 1090 Brussels, Belgium; 3 Center for Neurosciences (C4N), Vrije Universiteit Brussel, Laarbeeklaan 103, 1090 Brussels, Belgium; 4 Research Foundation-Flanders (FWO), Leuvenseweg 38, 1000 Brussels, Belgium; 5 Pain in Motion Research Group (PAIN), Department of Physiotherapy, Human Physiology and Anatomy, Faculty of Physical Education and Physiotherapy, Vrije Universiteit Brussel, Laarbeeklaan 103, 1090 Brussels, Belgium; 6 Charles E. Schmidt College of Medicine, Florida Atlantic University, 777 Glades Road, BC-71, Boca Raton, FL 33431, USA; 7 Department of Physical Medicine and Physiotherapy, Universitair Ziekenhuis Brussel, Laarbeeklaan 101, 1090 Brussels, Belgium; 8 Department of Public Health (GEWE), Faculty of Medicine and Pharmacy, Vrije Universiteit Brussel, Laarbeeklaan 103, 1090 Brussels, Belgium; 9 Laboratory for Brain-Gut Axis Studies (LaBGAS), Translation Research in Gastrointestinal Disorders (TARGID), Department of Chronic Diseases and Metabolism (CHROMETA), KU Leuven, Oude Markt 13, 3000 Leuven, Belgium; 10 Movement & Nutrition for Health & Performance Research Group (MOVE), Department of Movement and Sport Sciences, Faculty of Physical Education and Physiotherapy, Vrije Universiteit Brussel, Pleinlaan 2, 1050 Brussels, Belgium; 11 Department of Health and Rehabilitation, Unit of Physical Therapy, Institute of Neuroscience and Physiology, Sahlgrenska Academy, University of Gothenburg, SE-405 30 Gothenburg, Sweden; 12 Department of Biostatistics and Medical Informatics, Faculty of Medicine and Pharmacy, Vrije Universiteit Brussel, Laarbeeklaan 103, 1090 Brussels, Belgium; 13 Department of Radiology, Universitair Ziekenhuis Brussel, Laarbeeklaan 101, 1090 Brussels, Belgium

**Keywords:** electroencephalography, spinal pain, brain signature, pain perception, patient education

## Abstract

**Background/Objectives**: Biopsychosocial pain neuroscience education (PNE) has recently gained attention in preparing patients for surgery. PNE is expected to influence pain coping strategies and descending nociceptive inhibition. The goal of this study was to compare cortical evoked responses during experimental pain processing using a conditioned pain modulation (CPM) paradigm between patients receiving perioperative PNE (PPNE) or perioperative biomedical back school education (PBSE). **Methods**: This predefined EEG subgroup analysis included only participants with complete EEG recordings at baseline and 6 weeks. Of these, twenty-three patients with low back-related leg pain, scheduled for lumbar spine surgery, were randomized to either two sessions of PPNE or two sessions of PBSE. All patients were stimulated electrically at the median nerve of the symptomatic side and the sural nerve of the symptomatic and non-symptomatic side before and 6 weeks after the educational sessions, while evoked potentials were recorded by electroencephalography (EEG). Subsequently, this protocol was repeated during the application of the CPM paradigm by immersing the hand contralateral to the symptomatic side into cold water. **Results**: A significant decrease in the amplitude of the waveforms during CPM was found compared to the waveforms before CPM at the non-symptomatic sural nerve. No significant differences were found at the other test locations. For the waveforms of the CPM effect (subtracted waveforms), no significant treatment effects were revealed between the PPNE and PBSE groups. **Conclusions**: These exploratory findings suggest that PPNE was not associated with differential modulation of EEG evoked potentials during CPM compared with PBSE at 6 weeks post-surgery.

## 1. Introduction

Lumbar spine surgery is recommended for lumbar radicular pain when conservative treatments fail [1,2]. Nevertheless, chronic pain after spinal surgery remains common, with a pooled prevalence of 14.9% (95% CI from 12.38 to 17.76) [3]. Several risk factors for chronic post-surgical pain have been identified, including multi-level surgical procedures [4] and psychological factors [5]. In particular, negative psychological factors such as depression, pain catastrophizing and anxiety [5,6], unrealistic expectations regarding surgical outcomes, preexisting pain chronification and psychological disorders are related to an increased risk of persistent pain after surgery [7,8].

The interplay between persistent pain and psychological factors is well established [9]. To decrease risk factors for chronic post-surgical pain and to better prepare patients for surgery and postoperative rehabilitation, pain neuroscience education (PNE) has gained interest during the last decade [10]. PNE is an educational intervention that explains the biological processes underlying pain with the aim of reducing its perceived threat through reconceptualization and increased pain knowledge [11,12]. In the perioperative setting, PNE has been associated with improvements in pain catastrophizing, kinesiophobia, and quality of life [13,14].

In patients with fibromyalgia, PNE was able to activate brain-orchestrated endogenous analgesia, also referred to as descending nociceptive inhibition [15]. Conditioned Pain Modulation (CPM) is thought to reflect the efficiency of the descending nociceptive inhibitory pathways [16]. In patients with spinal pain, the descending nociceptive inhibitory pathways are suggested to be malfunctioning [17]. Preoperative measures of descending nociceptive inhibition have a predictive value for chronic pain following surgery [18,19]. Evaluation of brain oscillations via electroencephalography (EEG) can help unravel patterns of pain-related brain activity to assess whether PNE can influence cortical processes. Evidence directly linking PNE to central nervous system level changes is still emerging, with available reports suggesting potential neurobiological correlates, particularly when PNE is delivered in sufficiently intensive or multimodal formats [20]. Previously, a case report in a 30-year-old female patient with lumbar radiculopathy reported on functional MRI findings following PNE [21]. Compared with pre-education, a deactivation of the periaqueductal grey area, a deactivation of the cerebellum, and an increased activation of the motor cortex were revealed [21]. Additionally, a randomized controlled trial in patients with chronic nonspecific spinal pain showed that combined PNE and cognition-targeted exercise therapy led to a significantly higher supramarginal thickness on MRI compared with patients who received education on back or neck pain combined with general exercise therapy [22]. However, studies examining whether functional brain changes in patients with low back related leg pain improve in response to PNE are essentially lacking and represent an important knowledge gap.

For all these reasons, the present study aimed to examine whether PNE, compared with perioperative biomedical back school education, is associated with differences in cortical evoked potentials during a CPM paradigm in patients undergoing surgery for low back-related leg pain. We hypothesized that perioperative pain neuroscience education, compared with perioperative biomedical back school education, would be associated with enhanced modulation of cortical evoked potentials during conditioned pain modulation at 6 weeks after surgery.

## 2. Materials and Methods

### 2.1. Participants

Presurgical patients with low back-related leg pain, aged 18–65 years, were recruited from the Department of Neurosurgery at Universitair Ziekenhuis Brussel between June 2016 and April 2019. Only patients with low back-related leg pain with an indication for spine surgery were contacted to participate. An objective confirmation of an anatomical abnormality on neuroimaging was present, and this anatomical indication needed to be in line with the clinical presentation. Exclusion criteria were symptoms of spinal cord compression, chronic illness accompanied by uncontrolled chronic pain, rheumatoid, neurological, or psychiatric disorders, being pregnant, and pregnancy during the last year.

All patients provided written informed consent before participation. This study was conducted according to the revised Declaration of Helsinki (1998). The present analysis comprises a subgroup analysis of a multicenter randomized controlled trial, which was conducted in Universitair Ziekenhuis Brussel (Brussels, Belgium), AZ Dimpna (Geel, Belgium), and AZ Sint-Maarten (Mechelen, Belgium) [23]. The study protocol was approved by the Ethics Committee of the Universitair Ziekenhuis Brussel (B.U.N.143201526926) and registered on ClinicalTrials (ClinicalTrials.gov NCT02630732) on 15 December 2015.

### 2.2. Study Protocol

In total, patients underwent six assessments. One was scheduled in the week preceding surgery (baseline), and the remaining five follow-up assessments took place 3 days following surgery, 6 weeks (=primary endpoint), 6 months, 12 months, and 24 months post-surgery. Patients were randomized (1:1 ratio) to receive two sessions of perioperative PNE (PPNE) as experimental intervention or perioperative back school education (PBSE) as control intervention (the day before surgery and the second day post-surgery). As described in the published study protocol [24], pain and correlates of nociceptive processing (i.e., electrical detection and pain thresholds, temporal summation and conditioned pain modulation) were the primary outcome measures. In a subgroup of the sample (patients recruited at the Universitair Ziekenhuis Brussel), the conditioned pain modulation paradigm was supplemented by EEG recordings. This EEG supplementation was predefined and implemented prospectively at that site as part of the data collection procedures. The present study aimed to explore differences in evoked potentials between both educational interventions at baseline versus 6 weeks post-surgery (i.e., the primary endpoint).

Patients were aware of the intervention they received; however, they did not know whether this was the experimental or control intervention. Outcome assessors were blinded to the maximal extent possible. Randomization was performed by an independent investigator, using random permuted blocks of 4 to 6 weeks in which either the experimental or the control treatment was provided for a duration of one week, in random order (i.e., experimental week or control week). For the present EEG subgroup analysis, only participants with complete EEG recordings at baseline and at the 6-week follow-up were included. As a result, group sizes in this subgroup do not reflect the original 1:1 randomization ratio of the parent trial, but rather the availability of complete EEG data.

### 2.3. Interventions

Both interventions consisted of two individually tailored, one-to-one sessions provided by trained physiotherapists (one preoperative session and one postoperative session), and an educational booklet. The first educational session took place on the day before surgery [25] and the second 2 days post-surgery, each session lasting approximately 60 min. Patients received the educational booklet after the first educational session and were expected to read this booklet before the second educational session.

The experimental intervention consisted of PPNE in which the following topics were addressed: the characteristics of acute versus chronic pain, how pain becomes chronic (plasticity of the nervous system, modulation, modification, central sensitization, etc.), potential sustaining factors of central sensitization, the decision to have lumbar surgery, surgical experiences, environmental effects on nerve sensitivity and recovery after surgery, readdressing patient’s illness perceptions, and discussing the way of applying the knowledge into the patient’s daily life. A more detailed description of the content of the education in this specific population can be found elsewhere [12].

During PBSE (i.e., control intervention), on the other hand, the normal course of back pain, anatomy, physiology and biomechanics of the lumbar spine, the expected course of postoperative back and leg pain, the importance of self-care, principles for reducing back load during activities of daily living, professional and leisure time activities, and ergonomic advice were discussed [26,27]. More information regarding the interventions can be found in the published protocol [24].

PBSE was selected as an active control intervention because it reflects standard educational care commonly provided to patients undergoing lumbar spine surgery. In the perioperative setting, withholding patient education was considered ethically and clinically inappropriate, as education regarding anatomy, postoperative expectations, and self-management is part of usual care. Therefore, PBSE served as a pragmatic comparator that allowed evaluation of the added value of a biopsychosocial PNE approach beyond standard biomedical education.

### 2.4. Outcome Measurements

#### 2.4.1. Pain Intensity

A 100 mm Visual Analogue Scale (VAS) was used to assess peak pain intensity in the previous 24 h, lowest pain intensity in the previous 24 h, average and current leg pain intensity, and average and current low back pain intensity. All questionnaires were provided electronically to patients. Previous research indicated that there is no clinically relevant difference between the assessment of VAS scores between paper-based, laptop, and mobile phone platforms [28].

#### 2.4.2. Descending Nociceptive Inhibition

Descending nociceptive inhibition was measured with a CPM protocol at three different locations, namely, at the sural nerve of the symptomatic leg, at the median nerve ipsilateral to the symptomatic leg side and at the sural nerve of the asymptomatic leg. The felt pad electrode was placed on the skin overlying the median nerve, whereby the cathode was located 5 cm proximally from the wrist and the anodal electrode 3 cm distally from the cathode [29]. The electrode for stimulation of the sural nerve was placed 2 cm posterior to the lateral malleolus, at the innervation area of the sural nerve [30]. At first, electrical stimuli (constant current pulse train of 5 pulses at 250 Hz) were applied (Surpass LT Stimulator (EMS Biomedical, Korneuburg, Austria)) on three test locations to determine the electrical pain threshold (EPT). Stimuli were gradually increased by steps of 0.5 mA, starting from 0 mA, until the participant experienced an unpleasant sensation [31]. Measurements were performed three times with a 30 s interval, whereby the mean of the three measurements was used in further analyses [32]. CPM was induced with electrical stimulation as the test stimulus, and the cold pressor test with water at 12 °C was used as the conditioning stimulus. This temperature is sufficient to produce a painful response but is bearable for a few minutes [33]. In the first phase, patients received 20 electrical stimuli [34] at an intensity of 140% of the individualized EPT [35]. Stimuli were provided with an inter-stimulus interval of eight to twelve seconds. After the last stimulation, patients were instructed to provide an average pain intensity rating for the 20 electrical stimuli on a verbal numeric rating scale (VNRS) from 0 to 10, with 0 indicating no pain and 10 the worst pain imaginable [36]. In the second phase, patients received the same test stimulus (TS), accompanied by the conditioning stimulus (CS). Patients were instructed to submerge the hand of the non-symptomatic side into the cold water up to the wrist. After the last electrical stimulation, participants could withdraw their hand (approximately 3 min after the first electrical stimulus). Again, an average VNRS score was asked for the 20 electrical stimuli. After applying the CPM paradigm at one body site, the patient was allowed to withdraw the hand from the cold-water bath for three consecutive minutes. Next, the CPM protocol was repeated for the second and third body sites in a randomized order. Patients received no specific instructions regarding their attention, although all patients were instructed to close their eyes during the testing. The total score on the CPM effect was calculated as follows: ((VNRS score _TS_ − VNRS score _TS + CS_)/VNRS score _TS_). Total scores were converted into a categorical variable, whereby total scores ≤ 0 and total scores > 0 were, respectively, coded as the absence and presence of the CPM effect.

#### 2.4.3. EEG Recordings and Time-Domain Analysis

EEG recordings were performed during the CPM assessment (vide supra). All assessments were conducted in a quiet, lighted room with the same equipment. Participants were instructed to sit as relaxed and still as possible. Additionally, they were instructed to close their eyes, avoid blinking and avoid making small movements with their face. A scalp EEG (Sienna digital EEG, EMS Biomedical, Korneuburg, Austria) with a 32-channel Sn surface electrodes headcap was used (Headcap, EMS Biomedical, Korneuburg, Austria), following the standard 10/20 montage system. Two reference electrodes were located behind the ears (earlobe references). Impedances were evaluated and kept as low as possible. The EEG was sampled against the AFz ground electrode. The EEG signals were analyzed offline using Letswave 7.0 (https://www.letswave.org/). After applying a 0.3–30 Hz band-pass zero-phase Butterworth filter to the continuous EEG recordings, the signals were segmented into epochs extending from −500 to +1500 ms relative to stimulus onset. Epochs contaminated by eye movements or eye blinks were corrected by using an Independent Component Analysis (ICA) [37]. Epochs with amplitude values exceeding ±100 µV were rejected as these were likely to be contaminated by artifacts. Bad channels were interpolated with the three most neighboring electrodes. Denoised epochs were then baseline corrected (reference interval: −500 to 0 ms) and re-referenced to the average. Finally, for each patient, epochs were averaged according to the location and condition (TS or TS + CS). As such, separate averaged waveforms were computed for each patient, time point (pre- and post-surgery), test location (wrist, symptomatic leg and non-symptomatic leg) and stimulation phase (TS and TS + CS). Afterwards, we computed for each patient the different waveforms to assess the CPM effect by subtracting the waveforms without cold water application from the waveforms with cold water at each location.

The amplitudes of the entire difference waveforms were compared for each test location using point-by-point ANOVAs with time as a within-factor and treatment as a between-factor. These were performed separately for each location to evaluate the effect of time (pre- and post-surgery) and treatment group (PPNE versus PBSE). An in-depth explanation of the cluster-based approach can be found in van den Broeke et al. (2017) [38]. In summary, different waveforms were compared using point-by-point F-statistics and adjacent samples in time above the critical F-value for parametric two-sided tests were identified and clustered. Amplitudes of the entire waveforms during TS and TS + CS were evaluated with a paired point-by-point permuted *t*-test (2000 permutations). Based on the results of our previous study [39], we performed the tests only on the midline central electrode. Clusters were considered significant if sufficiently large (≥5 msec) and *p* < 0.01.

### 2.5. Statistical Data Analysis

Statistical analysis was performed in R Studio version 0.99.903. Only patients with EEG data for the baseline and 6 weeks post-surgery assessments were included in this subgroup analysis. Normality was controlled with the Shapiro–Wilk test and QQ-plots. Demographics between both treatment groups were compared with Chi-square tests, *t*-tests and Wilcoxon tests. Wilcoxon tests were used to evaluate VAS improvements between the two visits. For nonparametric between-group comparisons, effect sizes were quantified using rank-biserial correlations with corresponding 95% confidence intervals. For EEG outcomes, within-subject effect sizes were expressed as Cohen’s *dz* and derived from the corresponding *t* or *F* statistics, with 95% confidence intervals calculated using standard analytical approximations.

To evaluate the presence of a CPM effect over time in both treatment groups, odds ratios were calculated. The Cochran–Mantel–Haenszel test was calculated to evaluate conditional independence. The null hypothesis stated that there is conditional independence between time and the presence of a CPM effect, given the treatment.

The parent randomized controlled trial was powered for its primary clinical outcomes, and analyzed according to the intention-to-treat principle for its primary clinical outcomes. This EEG analysis was conducted as an exploratory, hypothesis-generating subgroup analysis restricted to participants with complete EEG data at both time points (on a per-protocol basis on complete-cases). As the EEG component was not the basis for the original trial sample size calculation, no a priori power calculation was performed for EEG endpoints.

### 2.6. Role of the Funding Source

This study was funded by the Applied Biomedical Research Program of the Agency for Innovation by Science and Technology, Belgium (IWT–TBM project No. 150180). The funder played no role in the design, conduct, or reporting of this study.

## 3. Results

### 3.1. Demographics

**Figure 1:**
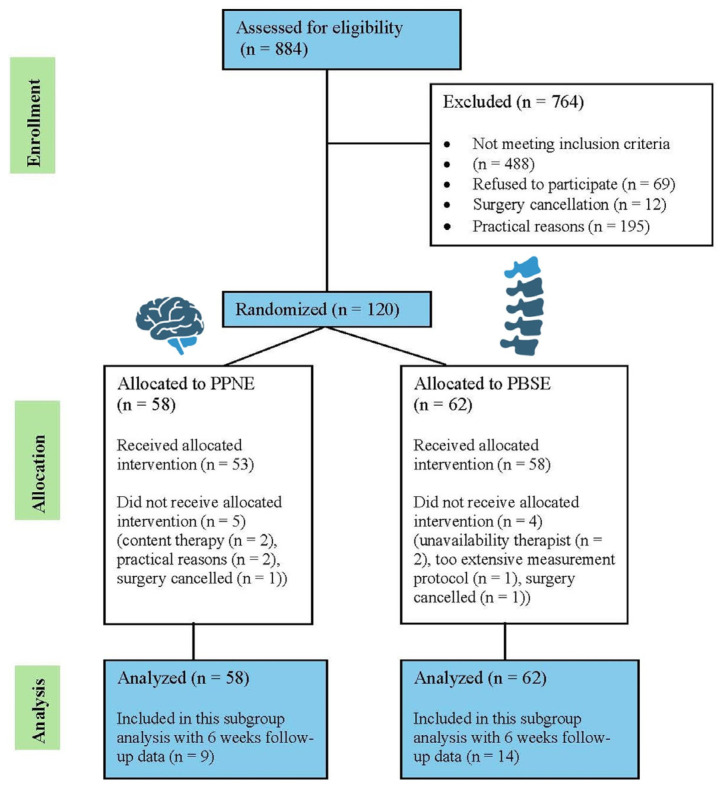
PRISMA flow chart for the RCT [23].

Although randomization in the parent trial was performed in a 1:1 ratio, the current EEG subgroup consisted of 23 participants with complete EEG data at both time points, resulting in unequal group sizes (PBSE n = 14; PPNE n = 9) (Figure 1).

No imputation procedures were applied for missing EEG data. No participants were excluded from this subgroup based on treatment allocation or outcomes. As such, twenty-three patients (15 males, eight females) were included. On average, patients were 47.3 (±11.8) years old. Fifteen patients (65%) were experiencing chronic pain, defined as pain present for three months or longer. The median VAS score for back pain intensity was 36/100 (Q1–Q3: 12.5–66) and 55/100 (Q1–Q3: 36–72) for leg pain intensity. No significant differences in baseline characteristics were revealed between the two treatment groups (Table 1).

### 3.2. Pain Intensity

At the visit around 6 weeks after surgery, the median VAS score for back pain intensity was 8/100 (Q1–Q3: 6–14) and 6/100 (Q1–Q3: 0.5–12) for leg pain intensity. Compared with baseline, a significant decrease in VAS score for back pain intensity (V = 229.5, *p* < 0.001) and leg pain intensity (V = 271, *p* < 0.001) was found. The median VAS improvement for back pain was 27/100 (Q1–Q3: 12–37) in the PPNE group and 21/100 (Q1–Q3: 1.75–57.75) in the PBSE group, without significant differences between the two groups (W = 63.5, *p* = 1). For leg pain intensity, the median VAS improvement was 54 (Q1–Q3: 28–58) in the PPNE group and 40.5/100 (Q1–Q3: 9.25–65.5) in the PBSE group, without significant differences between both groups (W = 68, *p* = 0.78). Between-group differences in pain improvement were negligible for both back pain (rank-biserial correlation r = 0.008, 95% CI −0.45 to 0.46) and leg pain (r = 0.08, 95% CI −0.39 to 0.52), indicating substantial overlap between the PPNE and PBSE groups.

### 3.3. Descending Nociceptive Inhibition

At baseline, 12 patients were unable to demonstrate a CPM effect on the wrist, while in 11 patients, this effect was shown. At the sural nerve on the symptomatic side and non-symptomatic side, 11 patients and 15 patients, respectively, showed a CPM effect. At the primary endpoint, 12 patients showed a CPM effect at the wrist, 11 patients at the symptomatic side and nine patients at the non-symptomatic side.

For all three test locations, the Cochran–Mantel–Haenszel test indicated conditional independence (meaning no association between time and CPM effect, conditional on treatment) (*p* > 0.05) (Table 2). When stimulating the wrist, a common odds ratio of 0.84 was found, meaning that a CPM effect was more often obtained at the visit after 6 weeks compared with the baseline visit. On the symptomatic side, a common odds ratio of 1 was found. On the non-symptomatic side, a CPM effect was more often obtained at the baseline visit compared with the visit after 6 weeks (common OR 2.92).

### 3.4. EEG Responses

**Figure 2:**
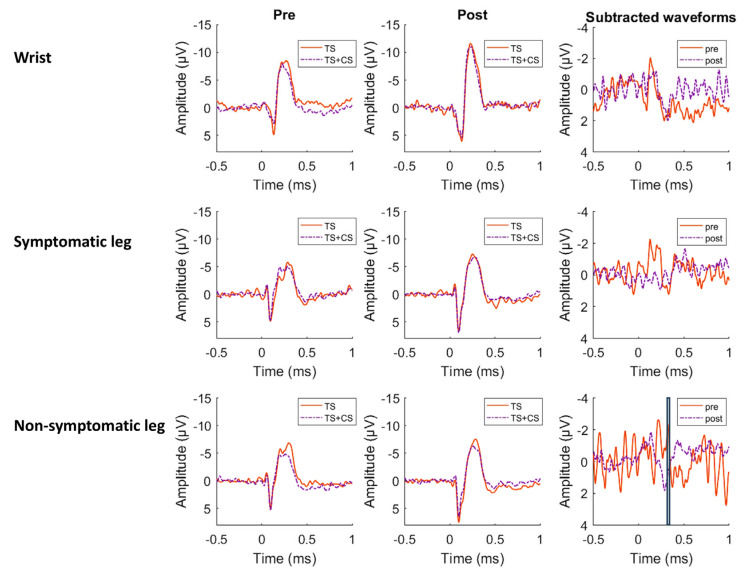
Group-level evoked potentials recorded at Cz, separated by stimulus location and time period (pre versus post-surgery). The different conditions of the CPM protocol are indicated by TS (test stimulus alone) and TS + CS (test stimulus + conditioning stimulus). In the third column, subtracted waveforms (TS + CS minus TS) are plotted. The black box shows the time window during which the two waveforms were significantly different (*p* < 0.01).

Grand average waveforms recorded at the vertex (Cz) electrode are presented in Figure 2.

**Figure 3:**
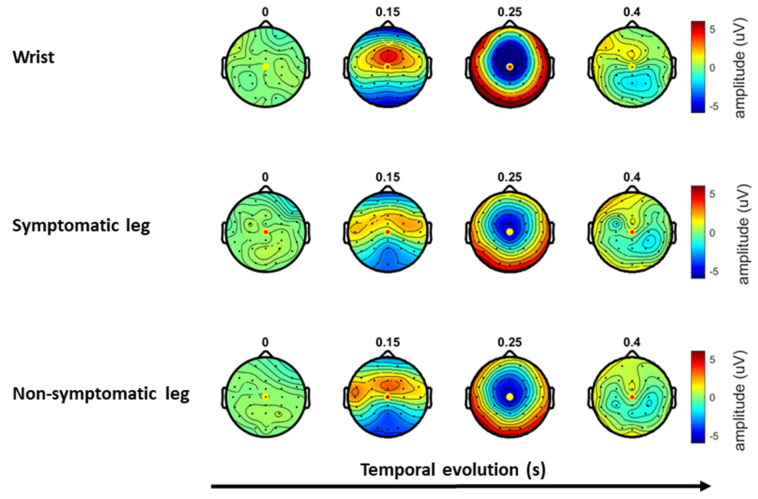
Scalp topographies for wrist, symptomatic and non-symptomatic leg stimulation, averaged over all patients. The temporal evolution of the averaged topography is expressed in seconds, from 0 to 0.4 s post-stimulus.

In all conditions (TS and TS + CS) and at each time, the electrical stimulus elicited a clear positive peak, followed by a more extensive negative peak. The magnitude of the evoked potential during TS + CS was considerably reduced as compared to the evoked potential during TS alone (Table 3). Scalp topography, separated by stimulus location, is presented in Figure 3. The positive peak is highest around the central electrode, with extension towards the temporal regions. The negative peak is highest at the central and frontocentral regions.

The positive peak of the grand-average evoked potential waveform elicited by stimulation of the wrist had an average amplitude of 7.84 µV (SD: 5.66 µV) with a latency of 112 ms (SD: 43 ms) post-stimulus. The negative peak was identified at a latency of 243 ms (SD: 43 ms) with an amplitude of −12.48 µV (SD: 5.77 µV). The positive peak of the waveform measured on the symptomatic leg had an average amplitude of 8.50 µV (SD: 6.52 µV) with a latency of 97 ms (SD: 37 ms), and the negative peak had an amplitude of −9.21 µV (SD: 4.68 µV) and a latency of 269 ms (SD: 60 ms). The positive peak of the waveform elicited by stimulation of the non-symptomatic leg had an average amplitude of 8.29 µV (SD: 5.89 µV) with a latency of 102 ms (SD: 38 ms), and the negative peak occurred at a latency of 260 ms (SD: 56 ms) with an amplitude of −9.31 µV (SD: 4.74 µV).

**Figure 4:**
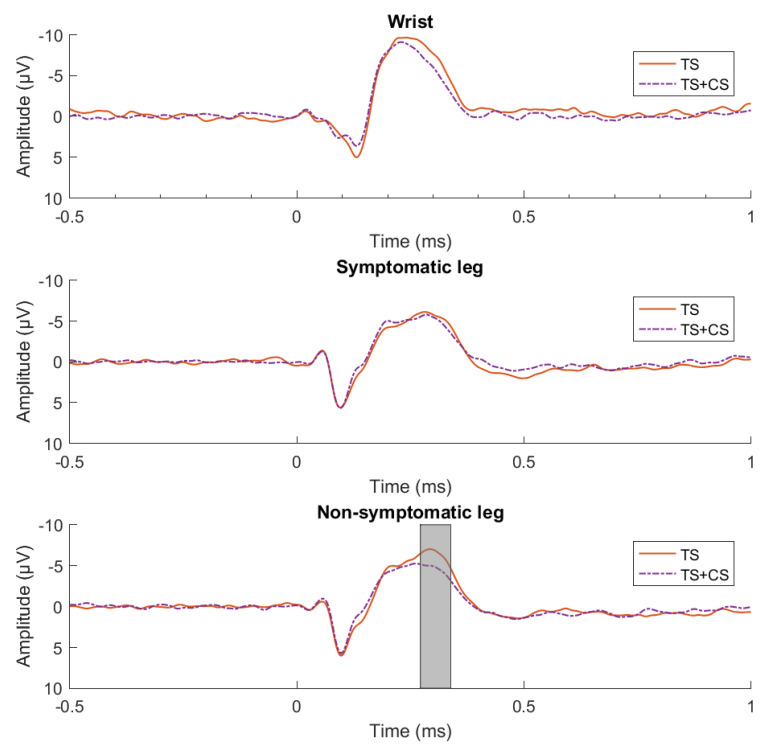
Group-level evoked potentials recorded at Cz, separated by stimulus location. The different conditions of the CPM protocol are indicated by TS (test stimulus alone) and TS + CS (test stimulus + conditioning stimulus). The grey box shows the time window during which the two waveforms were significantly different (*p* < 0.01).

Comparison of the waveforms after TS with the waveforms after TS + CS, performed with point-by-point permutation paired-*t*-tests at each location, revealed one significant cluster at Cz at the non-symptomatic side, extending between 272 and 339 ms post-stimulus (*p* < 0.01). In this cluster, the amplitude of the waveforms during TS was higher than the amplitude in the waveforms of TS+CS (t = 3.73, *p* = 0.002, Cohen’s dz = 0.78, 95% CI from 0.31 to 1.24) (Figure 4).

The results of the point-by-point ANOVA of the waveforms after stimulating the wrist and symptomatic leg could not determine any significant clusters. The point-by-point ANOVA of the waveforms after stimulating the non-symptomatic leg revealed one significant cluster at Cz, extending between 319 and 342 ms post-stimulus (*p* < 0.01). For this cluster, a significant difference was found between the pre- and post-measurement, whereby the amplitude in the waveforms at baseline was higher than in the measurement after 6 weeks (peak cluster value: F = 17.42, *p* < 0.001, Cohen’s *dz* = 0.87, 95% CI from 0.39–1.35). No significant time by treatment effects, nor treatment effects were found after stimulating the three locations. Overall, the results did not confirm the a priori hypothesis that PPNE would lead to greater modulation of cortical evoked potentials during CPM compared with PBSE.

## 4. Discussion

### 4.1. Summary of Main Findings

In this study, we extended the previously reported clinical outcomes of this randomized controlled trial [23] by comparing cortical evoked potentials during a CPM paradigm between patients who received PPNE and those who received PBSE. Electrical stimulation elicited clear evoked potentials in all conditions. The responses elicited by electrical stimulation were, on average, higher when stimulating the hand as compared to the foot (mainly due to the negative peak), a finding which is similar to the reporting of Truini et al. (2005) with laser stimulation [40]. A significant decrease in the amplitude of the waveforms during TS + CS was found compared with the waveforms of TS after electrical stimulation of the non-symptomatic sural nerve. No significant differences were found at the other test locations when comparing TS waveforms to TS + CS waveforms. When evaluating the waveforms of the CPM effect, no significant treatment effects were revealed between the waveforms of PBSE and PPNE. These findings indicate that PPNE, compared with PBSE, was not associated with differential modulation of EEG-derived evoked potentials during the CPM paradigm in this surgical population.

### 4.2. Interpretation of EEG and CPM Findings

Comparison of the waveforms during electrical stimulation and during descending nociceptive inhibition (electrical stimulation and cold-water immersion of the hand) did not reveal any differences when stimulation was provided on the wrist and symptomatic leg. This negative result is in line with the findings by Albu and Meagher (2019), where healthy participants received heat stimuli on the forearm before and during submerging the foot in noxious cold water [41]. The amplitude of contact heat-evoked potentials did not differ before and during the cold water immersion [41]. Additionally, this finding is in line with our previous reporting in healthy participants that the magnitude of responses, determined by a parallel factor analysis, during electrical stimulation of the median nerve and during electrical stimulation combined with cold water immersion of the other hand, did not differ [39]. Moreover, a systematic review could not reveal differences in ERP components between patients with chronic low back pain and healthy controls after electrical stimulation [42]. In the current study, only one significant cluster was revealed after stimulation of the non-symptomatic leg, whereby the waveforms of both phases to determine the CPM effect were different. This cluster, extending between 272 and 339 ms post-stimulus, indicated that the waveforms during CPM were attenuated immediately after the occurrence of the large negative peak at 260 ms post-stimulus. In patients with acute low back pain, electrical stimulation at the median nerve combined with an ice water application resulted in smaller event-related potential amplitudes than during the electrical stimulation alone in a time frame extending from 45 to 400 ms post-stimulus [43]. These findings indicate that there is still some controversy regarding similar or reduced event-related potentials during CPM, a finding that probably depends on the type of stimuli used to elicit CPM and the body location (remote versus affected regions).

In patients with various chronic pain disorders, PNE is an effective approach to change psychosocial factors and reduce healthcare expenditure [14,44]. Additionally, PNE has been shown to facilitate patients’ ability to cope with their condition [45,46] and has been associated with improvements in descending nociceptive inhibition in certain chronic pain populations [15]. In the published parent randomized controlled trial, PPNE resulted in more favorable outcomes in pain-related cognitions compared with PBSE, including greater reductions in kinesiophobia and pain catastrophizing, with effects already evident at the 6-week postoperative time point [23]. These findings support the notion that PPNE may primarily exert its effects through cognitive–affective pathways. However, the present findings suggest that such effects were not reflected in differential cortical evoked responses during CPM when PPNE was compared to PBSE in the perioperative context. In this study, one session was provided before surgery to provide patients with information on how to prepare for surgery, reduce anxiety and create positive expectations regarding the scheduled surgery [12,47]. As such, the aim is to facilitate effective pain coping. In opioid-treated chronic pain patients who received a Mindfulness-Oriented Recovery Enhancement intervention, associations were found between improvements in pain coping and increases in electrocortical responses [48]. However, in this study, no changes in cortical activity were detected in favor of PPNE. In line with the negative results on cortical activity, PPNE could not obtain higher pain intensity decreases than the control intervention. The total duration of the PPNE intervention may have limited the likelihood of detecting changes in evoked cortical responses, particularly given the brief and pragmatic nature of the perioperative educational exposure. Up till now, there is only limited evidence concerning brain alterations after PNE. A recent study evaluated gray matter adaptation in people with whiplash-associated disorders who received PNE combined with exercise compared with conventional physiotherapy [49]. Longitudinal whole-brain analysis revealed decreased gray matter volumes in the central operculum and supramarginal after treatment, not specific to either of the treatment modalities [49]. This result is in line with the higher supramarginal thickness on MRI in patients with chronic nonspecific spinal pain after combined PNE and cognition-targeted exercise therapy [22]. From a broader therapy intervention perspective, a meta-analysis evaluated functional brain changes on fMRI in patients who underwent psychotherapy sessions, whereby frontal, prefrontal regions, insular cortex, superior and inferior frontal gyrus, and putamen revealed longitudinal neural changes [50]. Additionally, a systematic review on neurobiological outcomes of cognitive behavioral therapy for obsessive-compulsive disorders revealed reduced activations in the orbitofrontal cortex, decreased activity in the striatum, increased activation in the cerebellum, and enhanced neurochemical concentrations in the anterior cingulate cortex, striatum and orbitofrontal cortex [51]. Based on brain alterations observed after psychotherapy and cognitive behavioral therapy interventions, it is expected that PNE (whether or not in combination with exercise programs) will result in long-term functional brain alterations in future explorations.

Despite the negative results regarding a treatment effect, one cluster captured a time effect, namely, a cluster extending from 319 to 342 ms post-stimulus after electrical stimulation at the non-symptomatic side. The amplitude of the CPM effect waveforms was attenuated after 6 weeks post-surgery compared with before surgery. When comparing evoked potentials, evoked by laser stimulation, an increased amplitude has been documented in patients with fibromyalgia [52,53] and patients with chronic tension-type headache compared to healthy controls [54]. It might be possible that the attenuated waveforms after surgery reflect a pain-relieving effect that is comparable to the state of healthy controls.

### 4.3. Clinical Implications

From a clinical perspective, the present findings suggest that while PPNE may improve psychological and patient-reported outcomes, such benefits are not necessarily accompanied by detectable changes in EEG-based measures of pain modulation in the early postoperative period. This distinction is important for clinicians, as it indicates that the absence of measurable neurophysiological effects should not be interpreted as a lack of clinical relevance or therapeutic value. Rather, perioperative educational interventions may primarily support patients by reducing threat, improving understanding, and facilitating adaptive pain coping during recovery. The findings also suggest that expectations regarding immediate neurophysiological changes following brief perioperative education should be tempered, and that education may best be viewed as one component of a broader, longitudinal biopsychosocial care strategy rather than a stand-alone intervention aimed at modifying cortical pain processing [46,55].

### 4.4. Methodological Considerations and Limitations

The intensity, duration, and timing of the PPNE intervention may also have influenced the present findings. The total PPNE exposure was limited to two sessions of approximately 60 min each, delivered immediately before and shortly after surgery. Although this dosage is pragmatic and consistent with perioperative clinical constraints, it may be insufficient to induce measurable changes in cortical pain processing, particularly in patients with chronic pain. Evidence from neuroimaging and electrophysiological studies suggests that detectable brain-related changes are more commonly observed following longer-lasting, repeated, or multimodal interventions, such as PNE combined with exercise therapy or cognitive-behavioral approaches delivered over several weeks or months [22,49]. For example, structural and functional brain adaptations have been reported after extended interventions involving repeated educational sessions and active behavioral components [49], whereas short-term educational interventions alone may primarily influence cognitive and psychosocial outcomes without immediately translating into detectable cortical electrophysiological changes. In addition, the perioperative period itself is characterized by acute nociceptive input, surgical stress, and pharmacological influences, which may further limit the detectability of subtle intervention-related cortical effects at early postoperative time points.

An additional methodological consideration relates to the operationalization of the CPM response. Although CPM is fundamentally a continuous construct, reflecting the magnitude of descending nociceptive inhibition, the CPM effect was analyzed as a binary outcome (presence versus absence) in the present study. This approach was chosen to enhance robustness and interpretability, given the limited sample size, and to align with previous clinical CPM studies using categorical classifications. However, dichotomization may reduce analytical sensitivity and obscure more nuanced modulatory effects on descending nociceptive inhibition. As a result, subtle changes in CPM related to perioperative educational interventions may not have been fully captured. Future studies with larger samples may benefit from analyzing CPM as a continuous measure to more sensitively detect intervention-related effects.

One of the limitations of this study is the lack of resting-state EEG evaluations before and after PPNE. Potentially, resting-state EEG might hold better properties to serve as a diagnostic biomarker for the development of chronic pain [56]. A previous study already denoted the potential value of higher theta and beta power in patients with chronic pain compared to healthy controls as a diagnostic biomarker [56] and even composite biomarkers consisting of band power and clinical data to predict outcomes are explored [57]. Another limitation is the limited subset of patients for whom EEG was available before surgery and 6 weeks post-surgery (23/120), which reduces statistical power and increases the likelihood of type II error, particularly for detecting small effects or effects that are spatially distributed across electrodes and time. Although our sample size is comparable to several CPM-related evoked potential/EEG studies [41,58], larger samples are likely required to robustly detect subtle cortical differences between educational interventions. Therefore, the absence of group differences in EEG-derived CPM responses in the present study should be interpreted cautiously and warrants replication in adequately powered cohorts. The use of an active educational comparator rather than a no-intervention control should be considered when interpreting the findings. While this design limits conclusions about the absolute effects of education per se, it strengthens the clinical relevance of the comparison by evaluating whether a PNE approach provides additional neurophysiological effects beyond standard perioperative education.

Another methodological consideration relates to the spatial restriction of the EEG analysis to the midline central electrode (Cz). This choice was based on prior work demonstrating that pain-related event-related potentials elicited by electrical stimulation show robust and reproducible responses at central midline electrodes [59,60]. Given the limited sample size and the exploratory nature of this subgroup analysis, the analysis was deliberately restricted to a priori defined ERP measures at Cz in order to minimize multiple testing and reduce the risk of false-positive findings. Nevertheless, restricting the analysis to a single electrode may have limited sensitivity to regionally specific cortical effects. Brain regions involved in cognitive–affective and modulatory aspects of pain processing, such as the anterior cingulate cortex, insula and prefrontal cortex, may not be optimally reflected in Cz-centered ERP measures. Consequently, more spatially distributed cortical effects related to perioperative educational interventions may not have been detected using the present ERP-based approach. In addition, the exclusive focus on time-domain ERP amplitude and latency captures stimulus-locked cortical responses but may be less sensitive to education-related adaptations expressed in non–phase-locked oscillatory activity or distributed functional interactions between brain regions. Such effects have been increasingly associated with cognitive and educational interventions and may, therefore, warrant complementary analytical approaches in future studies. Although pain cognitions were assessed, this EEG subgroup analysis did not examine correlations between psychological variables (e.g., kinesiophobia, catastrophizing, vigilance) and EEG outcomes. This was not a predefined aim, and the subgroup sample size limits the reliability of post hoc association testing. Finally, the timing of the post-intervention EEG assessment may also be relevant. EEG measurements were performed six weeks after surgery, corresponding to the primary endpoint of the parent trial and a clinically meaningful phase of postoperative recovery. Because EEG measurements were obtained six weeks after surgery, the present study cannot determine whether potential neurophysiological changes related to pain neuroscience education may occur at earlier perioperative time points or emerge over longer follow-up intervals. As such, the chosen assessment window may not have fully captured the temporal dynamics of cortical plasticity associated with perioperative educational interventions.

Finally, concomitant medication use was not systematically controlled for in the EEG analyses. In the perioperative setting, patients may receive a range of pharmacological treatments, including analgesics, opioids, gabapentinoids, and antidepressants, all of which are known to influence descending pain modulation and cortical electrophysiological activity. Concomitant medication use was not controlled for in the EEG analyses and may, therefore, represent a potential confounding factor.

Taken together, these results highlight that while perioperative educational approaches may influence psychological and clinical outcomes, detectable differences in EEG-based pain modulation measures between educational strategies are not necessarily observed within the early postoperative period, likely reflecting the methodological and contextual challenges inherent in capturing education-related cortical changes in a perioperative clinical population.

## 5. Conclusions

Electrical stimulation elicited clear cortical evoked potentials, with reduced amplitudes during conditioned pain modulation compared to test stimulation alone. However, PPNE did not result in differential modulation of EEG-based evoked potentials compared with PBSE in patients undergoing surgery for low back–related leg pain. Importantly, the absence of detectable neurophysiological differences should not be interpreted as evidence against the clinical or psychological value of pain neuroscience education, but rather as reflecting methodological and contextual factors, including the limited sample size, the sensitivity of the selected EEG outcome measures, and the timing of assessment in the early postoperative period.

## Figures and Tables

**Table 1:** Demographics of all patients, separated by treatment group. For age, the mean age is provided with standard deviation. For sex and the presence of chronic pain, exact counts and percentages are reported. For VAS leg and back pain intensity, median values with Q1 and Q3 are reported.

Variable	PBSE (N = 14)	PPNE (N = 9)	Test Statistic + *p*-Value
Age (years) ^a^	46.57 (±11.93)	48.33 (±12.33)	t(16.8) = 0.34, *p* = 0.74
Sex ^b^	11 (79%) males 3 (21%) females	4 (44%) males 5 (56%) females	χ2(1) = 1.5, *p* = 0.22
Chronic pain (pain > 3 months) ^b^	Present in 8 (57%) pts	Present in 7 (78%) pts	χ2 (1) = 0.32, *p* = 0.57
VAS back (/100) ^c^	30.5 (12.25–66)	36 (22–48)	W = 64.5, *p* = 0.95
VAS leg (/100) ^c^	52.5 (25.75–71.25)	62 (43–72)	W = 68, *p* = 0.78
CPM effect wrist ^b^	Present: 7 pts Absent: 7 pts	Present: 4 pts Absent: 5 pts	χ2 (1) = 0, *p* = 1
CPM effect symptomatic leg ^b^	Present: 6 pts Absent: 8 pts	Present: 5 pts Absent: 4 pts	χ2 (1) = 0.03, *p* = 0.87
CPM effect non-symptomatic leg ^b^	Present: 9 pts Absent: 5 pts	Present: 6 pts Absent: 3 pts	χ2 (1) = 6.3 × 10^−32^, *p* = 1

^a^ T-test; ^b^ Chi-square test; ^c^ Wilcoxon test. Abbreviations. PBSE: perioperative back school education; PPNE: perioperative pain neuroscience education; Pts: patients; VAS: visual analogue scale.

**Table 2:** Odds ratios for the presence of a CPM effect at the different visits, separated by treatment group and stimulation location.

	Treatment	Time	CPM Present	CPM Absent	OR [95% CI]	Common OR [95% CI]	M-H
Wrist	PBSE	Pre	7	7	0.75 [0.17 to 3.32]	0.84 [0.26 to 2.68]	χ2(1) = 0, *p* = 1
		Post	8	6			
	PPNE	Pre	4	5	1 [0.16 to 6.42]		
		Post	4	5			
Symptomatic leg	PBSE	Pre	6	8	1 [0.22 to 4.47]	1 [0.31 to 3.21]	χ2(1) = 0, *p* = 1
		Post	6	8			
	PPNE	Pre	5	4	1 [0.16 to 6.42]		
		Post	5	4			
Non-symptomatic leg	PBSE	Pre	9	5	3.24 [0.69 to 15.20]	2.92 [0.88 to 9.71]	χ2(1) = 2.09, *p* = 0.15
		Post	5	9			
	PPNE	Pre	6	3	2.5 [0.37 to 16.89]		
		Post	4	5			

Abbreviations. CPM: conditioned pain modulation; M-H: Cochran–Mantel–Haensel; OR: odds ratio; PBSE: perioperative back school education; PPNE: perioperative pain neuroscience education.

**Table 3:** Electrical evoked potential latencies and amplitudes separated by phase of the conditioned pain modulation protocol and location. The mean amplitude/latency is provided with standard deviation.

	PRE						POST					
	ERP Wrist		ERP Symptomatic Leg		ERP Non-Symptomatic Leg		ERP Wrist		ERP Symptomatic Leg		ERP Non-Symptomatic Leg	
	TS	TS + CS	TS	TS + CS	TS	TS + CS	TS	TS + CS	TS	TS + CS	TS	TS + CS
N amp (µV)	−12.21 (4.91)	−10.34 (4.45)	−9.07 (4.42)	−8.56 (4.13)	−9.72 (3.61)	−7.97 (3.57)	−14.22 (6.63)	−13.10 (6.45)	−10.30 (5.84)	−8.76 (4.35)	−10.03 (6.32)	−9.48 (5.08)
P amp (µV)	7.01 (5.12)	5.21 (4.34)	8.31 (6.24)	7.09 (5.10)	7.25 (5.05)	6.92 (4.65)	9.83 (6.70)	7.86 (5.58)	9.40 (7.72)	9.17 (6.93)	10.41 (7.19)	8.55 (6.07)
N lat (msec)	258 (45)	239 (43)	277 (66)	259 (67)	266 (56)	241 (63)	245 (43)	229 (32)	265 (47)	260 (46)	277 (43)	254 (51)
P lat (msec)	121 (47)	106 (49)	107 (45)	95 (35)	99 (40)	98 (35)	116 (32)	105 (43)	91 (34)	96 (36)	106 (39)	107 (40)

Abbreviations. Amp: amplitude, CS: continuing stimulus, ERP: event-related potential, lat: latency, N: negative peak, P: positive peak, TS: test stimulus.

## Data Availability

All data can be requested from the corresponding author based on reasonable request.
